# A Crowdsourced Topic Map and Future Research Agenda for Women’s Health

**DOI:** 10.2147/IJWH.S589408

**Published:** 2026-05-13

**Authors:** Stefaan Verhulst, Roshni Singh, Marta Dell’Aquila, Leonie Kunze, Cosima Lenz

**Affiliations:** 1 The Governance Laboratory, New York, NY, USA; 2 The Datatank, Tandon School of Engineering, New York, NY, USA; 3 Global Governance, Regulation, Innovation and Digital Economy Unit, Centre for European Policy Studies, Brussels, Belgium

**Keywords:** women’s health, question science, research framework, topic map

## Abstract

**Purpose:**

Women’s health research remains under-resourced, underprioritized, and narrowly defined. Across the life course, women experience distinct health needs with significant implications for health and wellbeing, yet persistent gaps in evidence and data continue to reinforce inequities. In the absence of a universally accepted definition of women’s health, this study aimed to develop a topic map to capture its breadth and to identify an evidence-informed set of the top ten priority questions to guide future women’s health research and innovation.

**Methods:**

We used a participatory, iterative methodology inspired by the 100 Questions Initiative, combining structured stakeholder engagement, rapid evidence synthesis, and iterative validation. An initial topic map was developed through an in-person workshop and refined through ongoing engagement with 77 global experts in women’s health and data science. Guided by the topic map, experts submitted research questions via a virtual survey, which were refined, clustered, prioritized, and ranked.

**Results:**

The topic map served as a shared framework to guide the submission of actionable research questions and comprised four branches: (1) key domains of women’s health; (2) determinants and barriers; (3) technology and innovation; and (4) research and evidence gaps. A total of 113 questions were submitted, clustered into 56 themes, and narrowed to a top ten through expert prioritization, followed by public ranking via a virtual survey that yielded 115 responses. The highest-ranked questions focused on reframing and prioritizing women’s health, strengthening investment and innovation ecosystems, and addressing evidence gaps, research participation, data quality, and equity.

**Conclusion:**

This study presents a comprehensive topic map that captures the complexity and cross-sectoral nature of women’s health and provides a unifying framework for the field. The prioritized questions offer a strategic foundation to guide future global research, policy, and investment to advance women’s health innovation.

## Introduction

Women comprise more than half of the world’s population and live longer than men, yet they spend a quarter more of their lives in poor health.^1^,^2^ Despite this, women’s health remains underfunded, underrepresented, and narrowly conceptualized, often confined to sexual and reproductive health.^3–5^ This narrow focus limits the global capacity to recognize and respond to the full spectrum of women’s health challenges across all stages of life.^6^

Women experience unique physiological and psychological transitions, including menstruation, pregnancy, childbirth, and menopause, each carrying distinct implications for health and wellbeing. Yet research into these experiences, and their links to disease, prevention, and treatment, remains insufficient.^7^ This includes menstruation, gynecological conditions such as endometriosis, and pregnancy-related health matters that remain underfunded and under-researched, contributing to inadequate understanding and insufficient and limited treatment options.^3^,^8^ Persistent gender bias in biomedical research and innovation continues to reinforce these gaps: women of reproductive age or pregnant women are often excluded from clinical trials, and medical knowledge still relies heavily on male physiology as the default.^3^,^5^,^9^,^10^ These inequities extend beyond research to the implementation of innovations, where gender norms, unequal digital access, and the gendered division of labor shape both participation and benefit.^11^,^12^

The growing pressures of global challenges such as climate change, antimicrobial resistance, and digital transformation further heighten the urgency for a renewed and more comprehensive understanding of women’s health.^13^,^14^ Women are often disproportionately exposed to these threats, making the integration of sex and gender perspectives across medicine, research, and public health essential. However, the absence of robust, sex-disaggregated data continues to impede evidence-informed policymaking and the design of interventions responsive to women’s lived realities.^15^

Advancing women’s health requires acknowledging that wellbeing is determined by intersecting social, economic, environmental, and biological factors that affect men and women differently.^16^,^17^ Improving women’s health is therefore not only a matter of equity; it is a cornerstone of stronger, more resilient health systems and a catalyst for sustainable development.^5^,^18^

There is still no universally accepted definition of women’s health or women’s health research. Definitions focus on conditions that occur only, mostly, or differently in women.^4^ A broader, more inclusive understanding is needed, one that reflects the diversity and complexity of women’s health across all stages of life.

To move toward this vision, a broader, more inclusive understanding of women’s health is needed, one that recognizes conditions that occur only, mostly, or differently in women while also accounting for how gender dynamics shape access to care, research priorities, and innovation. As part of the 100 Questions Initiative, this study presents the development of a topic map that characterizes the breadth and structure of women’s health as a global research and innovation domain, along with an evidence informed set of the top ten priority questions to guide a future agenda for advancing women’s health innovation.^19^

This study offers added value through the application of an innovative, crowdsourced methodology that leverages the expertise of individuals across the women’s health research field to collaboratively contribute on a more equal footing. This approach contrasts with traditional models that rely on consensus panels or top-down priority-setting processes. By incorporating a crowdsourced strategy, the study introduces a novel lens for data collection and prioritization, enabling more diverse input and broader engagement of relevant stakeholders.

## Methods

The development of the women’s health research agenda followed a participatory, iterative methodology inspired by the 100 Questions Initiative (100Qs) developed by the Governance Laboratory (The GovLab). The 100Qs methodology is a participatory approach that engages experts of a certain domain along with the public to identify and prioritizes the top actionable questions that can spur progress towards addressing complex societal challenges. The methodology is designed to identify priority research questions through structured stakeholder engagement, rapid evidence synthesis, and iterative validation, ensuring that the resulting agenda is evidence-informed and responsive to the needs of practitioners, policymakers, and researchers. This approach has been successfully applied across diverse global health domains to generate actionable, consensus-driven priorities including adolescent mental health.^20^ For women’s health, the 100Qs methodology was implemented in partnership with the Centre for European Policy Studies.

Our process comprised three sequential components: (1) development of a visual topic map of women’s health; (2) expert consultation and question development; and (3) final prioritization and ranking of questions. Ethical approval was not required as the study utilized anonymous, voluntarily submitted crowdsourced data with no personal identifiers.

### Development of the Women’s Health Topic Map

The initial topic map was created through an in-person workshop held in March 2025, convening 18 global experts in women’s health. Discussions were structured around the central question: “What falls under the spectrum of women’s health?” Participants identified and organized key elements into three overarching categories:

- Domains: Core thematic areas encompassed within women’s health.
- Challenges: Existing and emerging issues that hinder or could adversely affect women’s health.
- Innovations: Fields with potential to generate new discoveries, technologies, or transformative advances shaping the future of women’s health.

Using Miro, the project team visually mapped participant insights using these categories, capturing relationships between subtopics and identifying areas requiring further exploration. The topic map served as the first iteration of a comprehensive framework representing the scope and complexity of women’s health.

The map organizes topics in four categories: (1) key domains, (2) technology and innovation, (3) determinants and barriers, and (4) research and evidence gaps. These clusters were derived through thematic assessment of workshop discussions designed to capture the multidimensional nature of women’s health. Each main branch unfolds into a series of cascading sub-branches, reflecting the relative priorities identified across areas.

### Iterative Expert Consultation

The initial iteration of the topic map was presented in June 2025 to a broader group of 77 global experts in women’s health and data science during a virtual consultation. These experts represented 30 countries, with approximately half from high-income settings and half from low- and middle-income settings and included 66 women and 11 men. Experts represented diverse areas of expertise, including researchers, clinicians, practitioners, and advocates specializing in women’s health domains such as infectious disease, cardiology, mental health, femtech, fertility, and aging, among others. Participants were invited to review the map, identify omissions, recommend refinements, and comment on the relationships among domains, challenges, and areas of innovation. Their feedback informed the development of a revised second iteration of the map.

This iterative process aligned with the participatory principles of the 100Qs methodology, ensuring that the emerging agenda was crowd sourced, grounded in the perspectives of domain experts, and informed by current evidence and practice. The criteria guiding prioritization within the 100 Questions approach included:

- Desirability: Would investment in the topic transform understanding or generate high-impact interventions?
- Novelty: Is the topic poised to produce new insights or extend existing knowledge?
- Feasibility: Could meaningful progress be achieved in the near term if resources are allocated?
- Scalability: Can findings or interventions be applied across diverse contexts or populations?

## Results

### Women’s Health Innovation Topic Map

**Figure 1:**
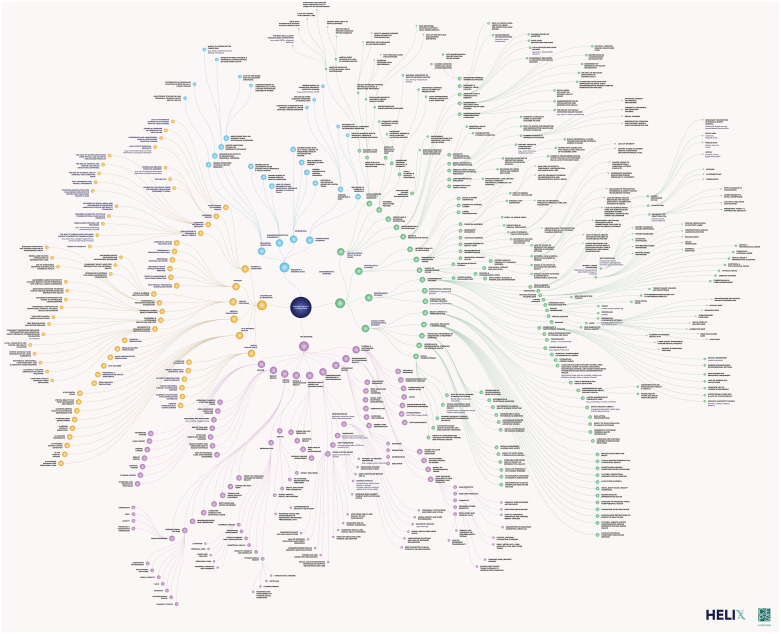
Women’s Health Topic Map. Color Key: Purple: key domains branch; green: determinants and barriers branch; yellow: technology and innovation branch; blue: research and evidence gaps branch.

The central themes in women’s health innovation were distilled into a visual topic map structured around four primary branches (Figure 1). Finalized in June 2025, this map served as the foundation shared with experts to guide the submission of actionable questions aimed at advancing women’s health innovation globally. The four main branches (key domains, determinants and barriers, technology and innovation, and research and evidence gaps) each encompass multiple subtopics, which are described in greater detail below.

#### Key Domains

Key domains identified include sexual and reproductive health, mental health, women’s health across the lifespan and again, cancer, chronic and autoimmune diseases, breast health, and environmental and occupational health. Together, these domains capture the biological, social, and structural factors influencing women’s health across the lifespan from adolescents under geriatrics.

#### Determinants and Barriers

This section maps the social, cultural, and systemic barriers that impact women’s health and women’s health innovation. It considers factors including gender norms, cultural expectations, and inequities that shape women’s ability to make informed health decisions and receive adequate care. The main branches include health equity and access, health systems policies and legal issues, gender-based violence, patriarchal gender norms, social connectedness, workplace and environmental stressors, biological differences in disease risk and treatment response, cultural gender and religious norms, and biases myths and misconceptions.

#### Technology and Innovation

The branch highlights the innovations and technologies transforming and posing barriers for women’s healthcare. It discusses advancements in digital health, AI, and FemTech, as well as challenges related to commercialisation, gendered pricing, and access to essential health services. The main sub-branches in this section include AI in women’s health, medical innovations, digital health tools, women as consumers, and health solutions.

#### Research and Evidence Gaps

This section maps women’s health research and depicts underrepresentation of women in clinical trials, the lack of gender-specific research, and the need for more targeted funding to address overlooked health conditions. The main subbranches in this section include underrepresentation in research, neglected conditions, gender bias, and underfunded research.

### Question Development and Prioritization

Using the topic map as a guiding framework, the 77 global experts were invited to submit at least one research question through an online survey, focusing on areas where further research and innovation are needed. For each question, experts were also asked to provide a brief rationale and to share any relevant published or grey literature supporting the proposed inquiry.

A total of 113 questions were submitted. Of these, of the 113 questions submitted, 30% (34 questions) were descriptive (what is), 38% (43 questions) were prescriptive (how), 16% (18 questions) were predictive (what will), and 16% (18 questions) were predictive (what will). Following submission, the project team reviewed all submitted questions to assess quality, identify overlaps, and consolidate similar entries to ensure that the resulting set was coherent, non-duplicative, and actionable. Questions with overlapping subject matter and focus were aggregated and combined as possible.

This process resulted in 56 refined and clustered questions organized across 15 thematic clusters. These clustered questions were then circulated to the expert group for review and prioritization. Table 1 presents the subtopics and the proportion of submitted questions associated with each thematic area.

**Table 1:** Thematic Subtopics

Subtopic	Percent of Submitted Questions Across Subtopics (n=56)
Reframing, Redefining & Prioritization of Women’s Health	4 questions (7%)
Investment, Funding & Innovation Ecosystems	4 questions (7%)
Evidence Gaps, Research Participation, Quality & Data Equity	6 questions (11%)
Health Systems Responsiveness & Access Barriers	3 questions (5%)
Maternal Health & Mortality	4 questions (7%)
Menstruation & Reproductive Transitions	4 questions (7%)
Transgender & Gender-Diverse Health	1 question (2%)
Gender-Based Violence, Norms & Social Dynamics	4 questions (7%)
Hormones, Biology & Underlying Mechanisms	3 questions (5%)
Digital Health & AI in Women’s Health	5 questions (9%)
Policy, Governance & Global Coordination	4 questions (7%)
Cancer & Chronic Disease Outcomes	3 questions (7%)
Longevity, Ageing & Frailty	4 questions (7%)
Mental Health & Well Being	3 questions (5%)
Fertility, Contraception & Sexual/Reproductive Health	4 questions (7%)

Common themes that cut across these subdomains included equity and inclusion, evidence and data gaps, regulatory frameworks and equitable governance, emerging health technologies, stage specific and life course needs, including transitions, health system barriers, social cultural norms and gender power dynamics, economic and investment models, sustainable financing, and psychosocial aspects of women’s health.

### Expert Review and Prioritization

Following their review of the 56 clustered questions, experts provided detailed feedback on clarity, scope, and relevance. The questions were then iteratively refined to incorporate this input. Experts were subsequently invited to identify their top ten priorities from the updated set using an online form following the aforementioned prioritization criteria including desirability, novelty, feasibility, and scalability. Their responses were aggregated to generate the prioritized list of expert selected priority questions.

### Public Voting

To broaden participation and extend the crowd sourced methodology, the project team launched a public voting phase based on the expert derived top ten questions. An electronic survey was disseminated through social media, women’s health events including the Innovation Equity Forum, and targeted outreach to women’s health organizations and networks via email, Facebook, WhatsApp, and LinkedIn. Public voting yielded 115 responses from individuals across more than thirty countries, including thirteen European countries, eight African countries, one Asian country, four Middle Eastern countries, two North American countries, and one country each from Oceania and South America. The resulting output produced the final ranked list of the top ten priority questions, presented in Table 2, with the highest ranked questions originating from the subtopics reframing, redefining and prioritizing women’s health, investment and innovation ecosystems, and evidence gaps, research participation, quality and data equity.

**Table 2:** Top Ten Questions

Ranking	Questions	Subtopics	Rationale
1	What structural, social, and policy factors perpetuate women’s disadvantages across health, political, and economic domains and what evidence-based transdisciplinary strategies can effectively dismantle these barriers?	Reframing, Redefining & Prioritization of Women’s Health	Reframing women’s health, including reproductive, perinatal, and postpartum care, as societal infrastructure is critical because the evidence shows that such investments generate measurable spillover effects on population health, equity, and intergenerational well-being. Research demonstrates that comprehensive reproductive, maternal, newborn, and child health interventions substantially reduce maternal and neonatal morbidity and mortality while improving life expectancy and quality of life when delivered as system-level investments rather than as fragmented services. Postpartum care is particularly consequential: systematic reviews find untreated maternal depression leads to adverse maternal functioning and impaired infant development, making early detection and treatment an urgent public health priority. Conceptualizing women’s health as infrastructure highlights these measurable outcomes, maternal survival, infant growth and cognition, mental health, and continuity of care, as essential components of collective resilience. The economic and social returns of prioritizing women’s health further justify its treatment as infrastructure. Studies from the IMF and global investment frameworks consistently show that improvements in women’s health translate into higher labor force participation, productivity, and GDP growth by reducing gender gaps and enabling efficient allocation of human capital. Access to reproductive health and family planning lowers fertility, reduces dependency ratios, and increases investments in children’s education and nutrition, effects that are quantifiable across generations. Beyond economics, better access to health infrastructure redistributes time and care burdens, enabling women’s fuller participation in civic, educational, and political life.
2	What measurable social, economic, and health outcomes are associated with prioritizing women’s health (including reproductive health and postpartum care) as shared societal infrastructure rather than an individual burden?	Reframing, Redefining & Prioritization of Women’s Health	Women’s persistent disadvantages across health, political, and economic domains are rooted in interlocking structural, social, and policy barriers that limit access to resources, representation, and decision-making power. Research demonstrates that gender inequities in health are not only the result of biological differences but are heavily shaped by social determinants, such as education, income, and exposure to violence, that systematically disadvantage women. These inequities are reinforced by institutional and policy frameworks that fail to account for women’s specific needs, from underrepresentation in clinical trials to inadequate maternity protections. Political science literature further shows that women’s exclusion from governance and policy spaces perpetuates cycles of disadvantage, as policies designed without gendered perspectives often fail to correct or may even exacerbate inequities. Identifying the structural drivers of these disadvantages is therefore essential to developing interventions that are not only medically sound but also socially transformative. At the same time, dismantling these barriers requires evidence-based, transdisciplinary strategies that cut across health systems, political institutions, and economic structures. Public health research emphasizes the need for gender-responsive policies such as universal health coverage that includes maternal and reproductive health services, as a foundation for equity. Economics and development studies highlight that closing gender gaps in education, labor force participation, and political representation produces measurable returns in growth, productivity, and governance quality. Transdisciplinary approaches that integrate perspectives from epidemiology, sociology, law, and political economy have been proposed to address entrenched disadvantages, since they allow for the simultaneous use of multiple levers such as health interventions, legal reforms, and empowerment initiatives.
3	Which emerging or underserved areas in women’s health including promising technologies and commercially viable but underfunded innovations warrant strategic investment to drive transformative breakthroughs, improve health outcomes, and advance health equity?	Investment, Funding & Innovation Ecosystems	Identifying emerging and underserved areas in women’s health is urgent because longstanding structural biases in biomedical research and financing have systematically neglected women’s health needs. Studies show that women remain underrepresented in clinical research, leading to diagnostic gaps, delayed treatments, and inadequate therapeutic development for conditions that disproportionately affect them. Critical domains such as menopause, gynecological cancers, maternal mental health, and autoimmune disorders continue to receive disproportionately low funding despite high disease burden. This neglect reflects the gender health innovation gap, where research and development pipelines have historically prioritized male health outcomes. As a result, potentially transformative innovations—from digital health tools tailored to menstrual and reproductive health to postpartum care platforms—remain underfunded and under-scaled despite growing evidence of their efficacy. Strategic investment in promising technologies and commercially viable innovations can yield both health and equity dividends. Analyses highlight the economic case: McKinsey Global Institute estimates that advancing gender health equity could add trillions of dollars to global GDP by improving productivity and reducing preventable morbidity. Women’s health technologies (“femtech”), though attracting increasing attention, still represent only a fraction of overall health innovation funding. Expanding investment in these areas would not only improve outcomes for women but also generate spillover benefits for families and communities, as women’s health is tightly linked to child health, workforce participation, and intergenerational well-being.
4	What financing instruments, funding models, or regulatory approaches could attract and sustain investment in women’s health research and development (R&D) including women-led innovations in underserved areas?	Investment, Funding & Innovation Ecosystems	Financing models and regulatory frameworks play a decisive role in shaping the trajectory of biomedical innovation, yet women’s health R&D remains structurally underfunded compared with its disease burden. Analyses of global health financing show that areas such as reproductive health, maternal morbidity, menopause, and gynecological cancers receive disproportionately low investment despite clear public health and economic returns. Scholars attribute this underinvestment to market failures, gender bias in venture capital, and insufficient regulatory incentives. Moreover, women-led innovations face additional barriers, with evidence showing that female entrepreneurs secure significantly less venture funding than their male counterparts, even when controlling for sector and business fundamentals. These financing inequities perpetuate gaps in innovation pipelines and limit the commercialization of promising technologies that could transform women’s health outcomes. Exploring innovative financing instruments and regulatory levers is therefore critical to attracting and sustaining investment. Recent analyses highlight the importance of strengthening gender-lens investing frameworks and supporting funds dedicated to women-led innovation ecosystems.
5	What strategies, methodologies, and types of evidence are most effective in identifying and closing gaps in women’s health research that address barriers to scaling innovations for underserved populations?	Evidence Gaps, Research Participation, Quality & Data Equity	Gaps in women’s health research persist because systemic biases in evidence generation, ranging from underrepresentation of women in clinical trials to limited funding for gender-specific conditions, have constrained the knowledge base needed to design scalable innovations. For decades, biomedical research defaulted to male bodies as the “norm”, producing diagnostic blind spots and therapeutic inequities that continue to affect women’s health outcomes. Moreover, underserved populations, including women in low- and middle-income countries, minority communities, and those in fragile health systems, remain largely absent from research agendas. Identifying and closing these research gaps requires rigorous strategies for mapping disparities, including the use of sex-disaggregated data, gender-sensitive indicators, and intersectional methodologies that capture how overlapping social determinants (eg. poverty, race, disability) compound health inequities. By asking what methodologies and evidence are most effective, this question emphasizes the importance of improving both the quantity and quality of women-centered evidence to ensure innovations are relevant and inclusive. Closing these gaps is not only a matter of scientific rigor but also of translational effectiveness: without robust evidence on women’s health needs and contexts, promising innovations cannot be scaled equitably. Global health frameworks further highlight that evidence must extend beyond biomedical outcomes to include social, economic, and equity indicators if scaling is to advance health justice.
6	How can public funders of research and innovation (R&I) ensure that biomedical studies consistently collect and report sex- and gender-disaggregated data and what proportion of current research meets this standard globally?	Evidence Gaps, Research Participation, Quality & Data Equity	The consistent collection and reporting of sex- and gender-disaggregated data in biomedical research is foundational for generating evidence that is generalizable, equitable, and clinically relevant. Historically, biomedical research has disproportionately recruited male participants and often failed to analyze outcomes by sex or gender, producing significant diagnostic and therapeutic blind spots. This underrepresentation contributes to misdiagnosis, adverse drug reactions, and inequitable health outcomes for women. To address these shortcomings, public funders of research and innovation (R&I) are uniquely positioned to set enforceable standards by requiring sex- and gender-disaggregated data in grant applications, peer review processes, and reporting guidelines. The introduction of policies such as the NIH’s “Sex as a Biological Variable” (SABV) mandate illustrates how funder-level requirements can drive meaningful change in research design and reporting. Yet despite progress, compliance remains uneven globally, and a large proportion of biomedical research still does not meet sex- and gender-disaggregation standards. Other global assessments highlight that funding incentives and reporting mandates vary widely by region, with many low- and middle-income countries lacking institutionalized frameworks to ensure gender equity in research practices. Closing this gap requires not only harmonized funder policies but also monitoring systems and capacity-building initiatives that make compliance feasible across diverse research contexts.
7	What strategies to engage men and boys through violence prevention, caregiving, and reproductive health are most effective in challenging harmful social norms and improving health outcomes for women and gender minorities especially in low- and middle-income countries?	Gender-Based Violence, Norms & Social Dynamics	Engaging men and boys in violence prevention, caregiving, and reproductive health is increasingly recognized as a critical strategy for advancing women’s and gender minorities’ health, particularly in low- and middle-income countries where entrenched social norms strongly influence behavior. Evidence shows that gender norms not only perpetuate intimate partner violence and limit women’s autonomy, but also reduce men’s participation in caregiving and reproductive decision-making, reinforcing cycles of inequality. Programs that adopt a “gender-transformative” approach, actively questioning and reshaping harmful constructions of masculinity, have demonstrated effectiveness in reducing violence against women and increasing support for equitable caregiving. Randomized controlled trials have documented that engaging men as allies in reproductive health and parenting shifts attitudes, reduces violence, and improves both maternal and child health outcomes. These findings underscore the value of addressing masculinity directly, rather than treating men as peripheral actors in gender and health initiatives. At the same time, scholars stress that strategies must be multi-level and context-specific, combining community mobilization, policy advocacy, and service integration to sustain impact. Systematic reviews highlight that interventions are most successful when they move beyond individual behavior change to transform institutional practices such as integrating male engagement in antenatal care, or embedding violence prevention curricula into schools and youth programs. Crucially, these strategies also improve outcomes for men and boys themselves, reducing risky behaviors, mental health burdens, and reinforcing healthier models of masculinity. For women and gender minorities, the benefits extend across domains: reduced exposure to violence, more equitable sharing of care work, and greater autonomy in reproductive health.
8	How might gaps in women’s health data affect the accuracy of artificial intelligence (AI) diagnostics and what strategies can ensure AI systems are designed to serve everyone, not just the statistical majority?	Digital Health & AI in Women’s Health	Estrogen and progesterone, though classically defined as reproductive hormones, exert wide-ranging effects on non-reproductive organs including the brain, skeletal muscle, adipose tissue, cardiovascular system, and gastrointestinal tract. Neuroscience research demonstrates that estrogens influence synaptic plasticity, neuroprotection, and cognitive function, while fluctuations in progesterone are linked to mood regulation and susceptibility to affective disorders. Similarly, both hormones play central roles in metabolism: estrogen contributes to insulin sensitivity, lipid regulation, and body fat distribution, while progesterone modulates appetite and gastrointestinal motility. Disruptions in these hormonal pathways are implicated in conditions such as Alzheimer’s disease, sarcopenia, metabolic syndrome, and irritable bowel disorders, underscoring the need to systematically investigate their extra-gonadal effects. Without such understanding, women face both diagnostic uncertainty and suboptimal care during life stages characterized by hormonal transition, such as perimenopause and menopause. Hormone therapy (HT), prescribed for menopausal symptoms or endocrine disorders, further complicates this picture because its systemic impacts remain incompletely characterized. Evidence suggests that estrogen therapy can improve bone density and muscle strength, reduce central adiposity, and exert neuroprotective effects, yet it may also increase risks for thromboembolism or certain cancers depending on dose, formulation, and timing. Progesterone and progestins, meanwhile, have complex interactions with the central nervous system and metabolism that are not yet fully disentangled. Clinical trials such as the Women’s Health Initiative revealed both the promise and risks of HT, but subsequent analyses emphasize that effects vary by age, health status, and individual biology.
9	What are the effects of estrogen and progesterone on non-reproductive organs such as the brain muscles, metabolism, and digestion; and how does hormone therapy influence these systems?	Hormones, Biology & Underlying Mechanisms	Artificial intelligence (AI) systems in health care rely on the quality and representativeness of the data on which they are trained, yet women’s health data remain sparse, fragmented, and underrepresented. Clinical datasets have historically over-sampled men and under-sampled women, leading to diagnostic algorithms that may misclassify or overlook female-specific symptoms, particularly in cardiovascular disease, autoimmune disorders, and pain conditions. Systematic reviews show that sex and gender disparities in biomedical data create measurable algorithmic biases, reducing the accuracy and safety of AI diagnostics for women. For example, studies of machine learning models for cardiac risk prediction have documented significant performance differences by sex, raising concerns that these technologies may reinforce inequities rather than alleviate them. Understanding how data gaps propagate into biased AI outputs is therefore central to ensuring that emerging diagnostic systems do not perpetuate long-standing structural inequities in women’s health research. At the same time, evidence indicates that algorithmic fairness requires deliberate design strategies that extend beyond simply increasing sample size. Scholars highlight the need for sex- and gender-disaggregated data collection protocols, bias audits, explainability tools, and participatory approaches that involve women, especially from underrepresented groups, in the design and evaluation of AI systems. Regulatory bodies and health technology assessment frameworks are also beginning to call for transparency standards and validation benchmarks that explicitly address demographic equity. Embedding these safeguards can help ensure that AI diagnostic tools not only achieve technical accuracy but also advance health equity by serving women and gender minorities as reliably as the statistical majority.
10	How can policy, public health, and innovation systems better integrate sex- and gender-based data to close the gap between female longevity and female quality of life in old age?	Longevity, Aging & Frailty	While women consistently outlive men in nearly every country, research shows that longer life expectancy does not translate into better health or quality of life in old age. Women spend more years living with chronic disease, disability, and multimorbidity, particularly conditions such as osteoporosis, dementia, arthritis, and frailty syndromes. This discrepancy, often described as the “male-female health-survival paradox”, reflects the fact that policy and innovation systems have historically measured success primarily through longevity metrics, while underinvesting in quality-of-life indicators. Without integrating sex- and gender-based data into health surveillance and innovation systems, structural blind spots persist: clinical trials often exclude older women, epidemiological surveys underreport gendered determinants of aging, and public health strategies fail to capture the compounded effects of caregiving, widowhood, and socio-economic disadvantage on late-life health. Integrating sex- and gender-based data into public health, policy, and innovation systems provides a pathway to closing the gap between female longevity and quality of life. Evidence from gerontology and health policy research emphasizes that sex-disaggregated data can uncover unique risk factors, such as differential responses to pharmacological therapies, higher rates of dementia among older women, and gendered access to social protection systems, that are otherwise obscured in aggregate analyses. Innovation ecosystems, including digital health tools and aging-related technologies, also often fail to account for the specific needs of older women, limiting their effectiveness and equity. Strengthening governance frameworks to mandate sex- and gender-disaggregated reporting, supporting longitudinal cohort studies of aging women, and embedding gender analysis into healthy aging policies are strategies shown to improve outcomes. By centering women’s quality of life in old age, this question draws attention to the urgent need for a shift in measurement and innovation priorities that align with the realities of aging populations.

## Discussion

This study demonstrates the value of a participatory approach that remains underused in the field of women’s health research and innovation. By leveraging a collaborative process informed by expert input and international engagement, we generated a demand-driven set of priorities for future research with substantial potential to guide both action and strategic investment. The final list of priority questions was derived through a two-step process. First, a topic map was developed to synthesize expert insights regarding key domains, challenges, and areas of innovation in women’s health. Second, international experts contributed via online discussions, Email exchanges, and virtual surveys to refine, validate, and prioritize these questions.

Our methodology contributes to advancing women’s health innovation in multiple ways. Beyond producing a structured and ranked set of research questions that can inform the decisions of policymakers, funders, and other stakeholders, the topic map offers an updated conceptualization of the women’s health landscape. It provides a holistic perspective that extends beyond the traditional focus on reproductive and sex-specific conditions, emphasizing the complex interactions between biological, social, and systemic factors. As such, the topic map serves as a practical tool for advocates, policymakers, donors, and researchers seeking to reassess prevailing assumptions and identify strategic areas for action. When used alongside complementary resources, such as the Women’s Health Opportunity Map, it can illuminate persistent gaps and emerging opportunities, supporting better alignment of research, policy, and investment. Involvement of global experts not only strengthened the accuracy and legitimacy of our findings but also facilitated the identification of shared priorities that can underpin future cross-sectoral collaborations. However, these findings should be interpreted as strategic guidance rather than definitive or exhaustive priorities, given that they are derived from a defined group of participants and reflect a time-bound, participatory consensus process.

Several limitations warrant consideration. Although this work aimed for broad international representation, the number of participating experts was relatively small and disproportionately drawn from Europe and North America. This composition may have influenced both the feedback shaping the topic map and the outcomes of the prioritization process. Future iterations should seek to ensure voices that were underrepresented in this initial phase are meaningfully engaged, particularly from the Global South, Latin and South America, and individuals with diverse lived experiences across the life course. Repeated application of this methodology will also be necessary to ensure that research priorities remain current in a rapidly evolving women’s health landscape.

## Conclusion

Through an iterative and expert informed process, this study produced a topic map that offers a comprehensive conceptual framework for understanding the breadth and complexity of women’s health providing strategic priorities for this given moment. This framework served as the basis for identifying and ranking the top ten priority questions that can guide future research, policy development, and strategic investment. Together, the topic map and prioritized questions provide a structured foundation for advancing a more impactful women’s health research and innovation agenda. Operationalization of this research agenda will require sustained, cross-sectoral, and multilevel collaboration, alongside long-term investment, and institutional and political commitment.

## Abbreviations

100Qs, the 100 Questions Initiative; The GovLab, the Governance Laboratory; HI, Hormone therapy; AI, Artificial Intelligence.
